# An important role for adenine, cholera toxin, hydrocortisone and triiodothyronine in the proliferation, self-renewal and differentiation of limbal stem cells *in vitro*

**DOI:** 10.1016/j.exer.2016.09.008

**Published:** 2016-11

**Authors:** Min Yu, Sanja Bojic, Gustavo S. Figueiredo, Paul Rooney, Julian de Havilland, Anne Dickinson, Francisco C. Figueiredo, Majlinda Lako

**Affiliations:** a Institute of Genetic Medicine, Newcastle University, UK; b NHS Blood and Transplant, UK; c Institute of Cellular Medicine, Newcastle University, UK; d Department of Ophthalmology, Royal Victoria Infirmary, Newcastle University, UK

**Keywords:** Limbal stem cells, Limbus, Good manufacturing practice, Adenine, Cholera toxin, Hydrocortisone, Triiodothyronine, LSCs, limbal stem cells, LSCD, limbal stem cell deficiency, HAM, human amniotic membrane, CTX, cholera toxin, GMP, good manufacturing practice, EGF, epidermal growth factor, NHSBT, NHS blood and transplant, CFE, colony forming efficiency

## Abstract

The cornea is a self-renewing tissue located at the front of the eye. Its transparency is essential for allowing light to focus onto the retina for visual perception. The continuous renewal of corneal epithelium is supported by limbal stem cells (LSCs) which are located in the border region between conjunctiva and cornea known as the limbus. *Ex vivo* expansion of LSCs has been successfully applied in the last two decades to treat patients with limbal stem cell deficiency (LSCD). Various methods have been used for their expansion, yet the most widely used culture media contains a number of ingredients derived from animal sources which may compromise the safety profile of human LSC transplantation. In this study we sought to understand the role of these components namely adenine, cholera toxin, hydrocortisone and triiodothyronine with the aim of re-defining a safe and GMP compatible minimal media for the *ex vivo* expansion of LSCs on human amniotic membrane. Our data suggest that all four components play a critical role in maintaining LSC proliferation and promoting LSC self-renewal. However removal of adenine and triiodothyronine had a more profound impact and led to LSC differentiation and loss of viability respectively, suggesting their essential role for *ex vivo* expansion of LSCs. Replacement of each of the components with GMP-grade reagents resulted in equal growth to non-GMP grade media, however an enhanced differentiation of LSCs was observed, suggesting that additional combinations of GMP grade reagents need to be tested to achieve similar or better level of LSC maintenance in the same manner as the traditional LSC media.

## Highlights

• Adenine, cholera toxin, hydrocortisone and triiodothyronine are essential for LSCs proliferation and self-renewal.
• Adenine and triiodothyronine had a more profound impact as their removal led to LSC differentiation and loss of viability.
• Removal of each of four components from traditional culture media may pose a risk for clinical translations.
• New media composition in compliance with Good Manufacturing Practice is proposed.

## 1 Introduction

The major function of the cornea is to enable the transmission and focussing of light onto the retina at the back of the eye for visual perception (Adler and Hart, 1992). The presence of an intact limbal epithelium is essential for maintaining corneal transparency by acting as a barrier that prevents the conjunctival epithelium and its stromal blood vessels from encroaching onto the cornea (Chen and Tseng, 1991). The limbal epithelium also harbours the limbal stem cells (LSCs) which proliferate and differentiate to provide a lifetime source of corneal epithelial cells (Daniels et al., 2001, Dua and Azuara-Blanco, 2000, Sangwan et al., 2003). Loss of LSCs results in limbal stem cell deficiency (LSCD), characterized by the loss of corneal epithelial transparency and cyto-architecture, with consequent recurrent/persistent epithelial defects, encroachment of conjunctiva and its blood vessels onto the central cornea, deep corneal scarring, ocular surface inflammation, constant pain, poor vision and photosensitivity (Djalilian et al., 2003, Espana et al., 2002, Morgan, 2005, Pauklin et al., 2009, Puangsricharern and Tseng, 1995).

The patients with total and severe LSCD have been successfully treated with autologous limbal tissue transplants taken from the healthy contra-lateral eye (unilateral cases) or allogeneic transplants obtained from living related or cadaveric donors (unilateral and bilateral cases) (Kenyon and Tseng, 1989). Importantly, improvements in the *ex vivo* expansion of LSCs obtained from the culture of small limbal biopsies and the successful reversal of LSCD upon their transplantation has revolutionized the field and has reduced the risk to the donor eye, making this a widely used technique for treatment of LSCD in humans (Daya et al., 2005, Kolli et al., 2010). The *ex vivo* expansion of limbal epithelium prior to clinical transplantation, however, is still a relatively new technique, and as such, optimization and constant evaluation of the culture medium components are required for minimizing any risk to patients. The traditional culture media used by our group and others for the *ex vivo* expansion of limbal biopsies on human amniotic membrane (HAM) includes hydrocortisone, triiodothyronine, adenine and cholera toxin (Kolli et al., 2008, Meller et al., 2002, Pellegrini et al., 1997, Tsai et al., 2000). It is reported that hydrocortisone is important for maintaining distinct epithelial colonies as well as keratinocyte proliferation (Rheinwald and Green, 1975). Triiodothyronine is a hormone that has been proved useful in the cultivation of keratinocytes by reducing the requirement for fetal calf serum in epithelial cultures to minimal levels (Hayashi et al., 1978). Cholera toxin (CTX) is a protein complex secreted by the bacterium *Vibrio cholerae* and is responsible for the profuse, watery diarrhoea characteristic of cholera infection. It has been reported that CTX strongly stimulates colony growth from a small number of cultured human epidermal keratinocytes. The effect of cholera toxin on proliferation of keratinocytes has been associated with increased intracellular cyclic AMP level (Okada et al., 1982), whilst the addition of adenine to the culture media improves the colony forming ability of epithelial cells (Allen-Hoffmann and Rheinwald, 1984, Flaxman and Harper, 1975). However their individual contribution for the expansion and differentiation of LSCs in this culture system has not been examined in detail. The aim of this study, was to examine their individual roles on the growth rate, proliferation, viability and LSC maintenance during the *ex vivo* expansion of limbal explants on HAM and their possible replacement with Good Manufacturing Practice (GMP) grade reagents wherever possible. With this in mind Solu-Cortef^®^ (hydrocortisone sodium succinate) was used as hydrocortisone replacement, Actrapid^®^ (human insulin produced in *Saccharomyces cerevisiae*) as insulin replacement, Liothyronine (liothyronine sodium) as triiodothyronine replacement, Isoprenaline (L-isoproterenol) as cholera toxin replacement together with GMP-grade epidermal growth factor (EGF). Solu-Cortef^®^, Actrapid^®^, Liothyronine and Isoprenaline are commonly used drugs, and whilst the first three are direct replacements of the non-GMP certified components with the same chemical structure and effect, Isoprenaline has been chosen to replace cholera toxin since it stimulates proliferation of corneal epithelial cells using similar mechanism as cholera toxin by increasing the cyclic AMP levels (Reinach et al., 1992, Akhtar and Choi, 1994).

Our data suggests that all four components were essential for maintaining LSC proliferation and self-renewal. Replacement of each of the LSC media components with GMP grade reagents resulted in equal growth to non-GMP grade media; however an enhanced differentiation of progenitor cells in the outgrowth situated closest to the explant was observed, suggesting that additional combinations of GMP grade reagents need to be tested to achieve similar or better level of LSC maintenance in the same manner as the traditional LSC media.

## 2 Materials and methods

### 2.1 Human donor tissue

Cadaveric adult human limbal tissue was obtained from the corneo-scleral rings remaining (n = 7) after removal of the central cornea for transplantation in humans supplied by the NHS Blood and Transplant (NHSBT) Cornea Transplantation Service eye banks based in Bristol and Manchester, UK. Human amniotic membranes (HAM) were provided as individual units of 3 × 3 cm^2^ mounted on nitrocellulose paper by NHSBT, Tissue Services. Informed consent was obtained for research use of all human tissue.

### 2.2 Minimal medium and limbal explant culture

**Fig. 1:**
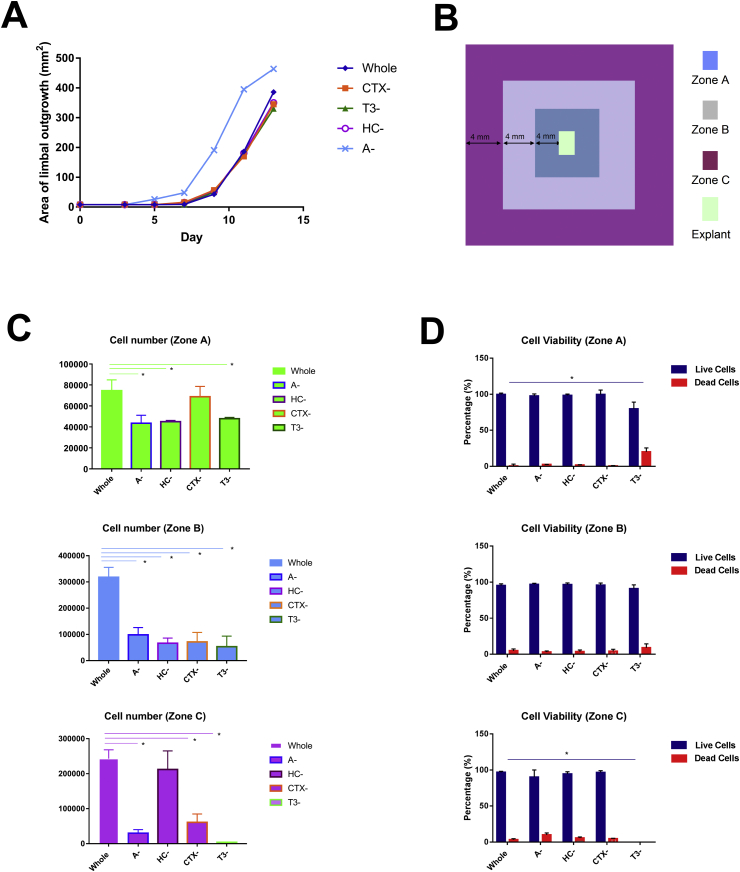
**Limbal epithelial cell growth and viability in whole and minimal media combinations**. (**A**) Schematic graph showing the area of explant outgrowth (mm^2^) on different days of culture. Whole: the explant cultured in whole medium; Explant- CTX-: the explant cultured in medium without cholera toxin; T3-: the explant cultured in medium without triiodothyronine; HC-: the explant cultured in medium without hydrocortisone; A-: the explant cultured in the medium without adenine; (**B**) Schematic presentation of explant outgrowths and their position into zones A, B and C relative to the limbal explant; (**C-D**) Assessment of cell numbers (**C**) and viability (**D**) of epithelial outgrowths in different media. (**A, C, D**) Data are presented as mean ± SEM, n = 3, * p < 0.05.

Limbal explant culture was performed as described by Kolli et al., 2010. In brief, the HAM was defrosted at room temperature in a class II laminar flow hood and washed twice with phosphate-buffered saline (PBS) (Gibco, UK) containing 1% penicillin/streptomycin and once with culture medium. Then the HAM was trimmed and wrapped around a sterile 24 × 24 mm^2^ glass coverslip with the epithelial side facing up and the overhanging part being folded over the edges of the coverslip. The HAM and its associated coverslip were placed on the top of a second sterile glass coverslip to lock the HAM in place and finally the whole construction was placed in a tissue culture well. Five limbal explants were prepared from each of three corneo-scleral rings (3 males, average age 67, SD 6.93, range 12), which had been stored in organ culture (supplied for corneal transplantation). The stromal tissue and deeper layers of the corneo-scleral rings were dissected away together with excess sclera leaving a ring containing approximately 2 mm of peripheral cornea and 2 mm of adjacent sclera, thereby including all the corneo-scleral limbus. Each ring was then divided into separate 4 mm^2^ segments and one of each such segment was carefully placed at the centre of the prepared HAM with a slight pressure for a few second to facilitate adhesion. Complete epithelial medium was prepared according to a previously validated composition: low-glucose Dulbecco's modified Eagle's medium (DMEM) 75% and Ham's F12 medium 25% (both Gibco, UK). This composition was supplemented with the following: fetal calf serum 10%, penicillin/streptomycin 1% (both Gibco, UK), hydrocortisone 0.4 μg/ml, insulin 5 μg/ml, triiodothyronine 1.4 ng/ml, adenine 24 μg/ml, cholera toxin 8.4 ng/ml and EGF 10 ng/ml (all Sigma-Aldrich, UK). The minimal media were prepared the same way as the complete epithelial medium with the exception that hydrocortisone, triiodothyronine, adenine or cholera toxin were removed as single components to study their impact on LSC self-renewal, proliferation and differentiation. Complete epithelial medium or minimal media were slowly added to ensure the explants were covered in medium without causing them to detach from the HAM (1.5 ml of medium). The medium was changed on the third day and then every other day thereafter. All cultures were carried out under identical conditions and placed in a tissue culture incubator at 37 °C with a humidified atmosphere containing 5% CO_2_. The epithelial outgrowths from each explant were marked at every medium change for the full length of the culture to allow comparison of growth rates. The cultures were terminated prior to the growth reaching the edge of the slides (at ∼14 days) and the epithelial outgrowth was divided into three equal zones (4.0 mm from the edge of the biopsy or the adjacent zone) as described by Kolli et al., 2008 and shown in Fig. 1B (Kolli et al., 2008). These three zones were used for analysis purposes: zone A indicating growth adjacent to the explant, zone B indicating intermediate outgrowth and zone C indicating the growth furthest away from the explant (Fig. 1B). At the end of the culture time, the epithelial cells from these zones were released and detached from the underlying HAM by 0.05% trypsin treatment for 10 min at 37 °C. The three samples of LSC-containing epithelial cell populations were then analysed for any differences in stem cell properties including colony-forming efficiencies (CFEs), immunocytochemistry and the expression of putative LSC markers by qRT-PCR.

### 2.3 Explant culture on GMP-grade medium

Two limbal explants were prepared from each of four corneo-scleral rings (3 males and 1 female, average age 62, SD 5.44, range 13), which had been stored in organ culture (supplied for corneal transplantation). The limbal explants were expanded in the GMP facility either in previously described standard non GMP-grade complete medium described above (n = 4) or new GMP-grade complete medium (n = 4) using HAM as a substrate. GMP-grade complete medium contained 75% of DMEM and 25% Ham's F12 medium supplemented with human serum 10% (Sigma-Aldrich, UK), penicillin/streptomycin 1% (Gibco, UK), Solu-Cortef^®^ 0.4 μg/ml (Pharmacia Limited, UK), Actrapid^®^ 5 μg/ml (Novo Nordisk, Denmark), Liothyronine 1.4 ng/ml (Mercury Pharmaceuticals Ltd., UK), Isoprenaline 2 μg/ml (South Devon Healthcare, UK), adenine 24 μg/ml (Sigma-Aldrich, UK) and GMP-grade EGF 10 ng/ml (Miltenyi Biotec, UK). Penicillin/streptomycin was removed from culture medium after the first 3 days of culture, as per our GMP protocol, in accordance with the MHRA. In addition, 4 different complete media were prepared with replacement of just one ingredient in isolation (Solu-Cortef^®^, Actrapid^®^, Liothyronine or Isoprenaline) to assess the impacts of individual replacements with GMP grade ingredients. Culture medium was changed at the third day and every other day thereafter. Expansion of each outgrowth was marked at every change of medium to allow monitoring of growth rates. Upon reaching 90% confluence the outgrowth were divided into three zones depending on proximity to the explant (A zone - inner, B - middle and C - outer zone) and cells were released from HAM as described above.

### 2.4 Cell counting and viability

20 μL of cells were aseptically transferred to a 1.5-mL clear Eppendorf tube and incubated for 3 min at room temperature with an equal volume of 0.4% (w/v) trypan blue solution prepared in 0.81% NaCl and 0.06% (w/v) dibasic potassium phosphate. Cells were first counted using a dual-chamber haemocytometer and a light microscope. A second flow cytometric method Tali Apoptosis Kit (A10788) which allows detection of apoptotic and necrotic cells using Annexin V and Propidium iodide staining was carried out to assess cell viability. This method also enables measurement of cell size.

### 2.5 Colony-forming efficiency assay

Colony-forming efficiency assay (CFE) assay is a method for determining the ability of the limbal epithelial progenitor cells to form colonies and assess their frequency. Mitotically inactivated J2–3T3 mouse fibroblasts were suspended in 3T3 medium: high-glucose DMEM (89%), fetal calf serum (10%) and penicillin/streptomycin (1%) and plated in a 9.6 cm^2^ tissue culture well at a final density of 2.4 × 10^4^ cells per cm^2^ and placed in a tissue culture incubator overnight to allow the establishment of a 3T3 feeder layer. The following day, 500 viable limbal cells from the limbal epithelial culture of interest were plated onto the prepared 3T3 feeder cells together with 2 ml of epithelial medium. The CFE culture was then placed in the tissue culture incubator and the epithelial medium was changed on the third day and then every second day thereafter with regular microscopic examination (Eclipse TS100, Nikon, Japan) for LSC colonies. The CFE was measured on the 12th day of the culture. This was performed by removal of the epithelial medium followed by two brief washes with PBS. The culture was then fixed with 3.7% formaldehyde (VWR International, UK) in PBS for 10 min at room temperature. Next, the formaldehyde solution was removed and the culture was irrigated with PBS. The colonies were then stained by incubation with 1% Rhodamine B (Sigma-Aldrich, UK) in methanol for 10 min at room temperature. Following staining, the colonies were counted under dissecting microscope (SMZ645, Nikon, Japan). The CFE was calculated using the formula: number of colonies formed/number of cells plated × 100.

### 2.6 Cytospin and immunocytochemistry

**Suppl. Fig. 1:**
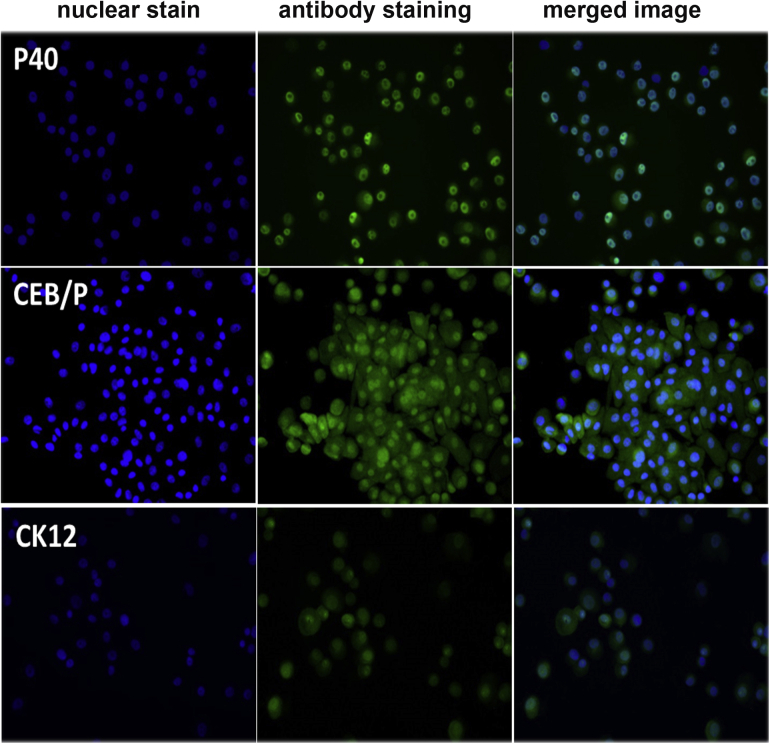
A representative example of immunofluorescent staining of cells harvested from outgrowth zone A with putative LSC and corneal epithelial cell markers performed at day 14 of explant culture on HAM using whole non-GMP grade media.

Cells were prepared by cytospin with cytocentrifugation speed at 1000*g* which provided a better cell distribution using a cytocentrifuge obtained from Shandon Southern Instruments, Sewickley, PA, USA. Immunocytochemistry was performed as previously described (Polak et al., 1975). Briefly, cells were fixed with 4% paraformaldehyde, permeabilised with 0.25% Triton X-100 (Sigma-Aldrich, UK), blocked with 5% BSA for 1 h, and incubated with primary antibodies including anti-delta NP63 antibody, P40 (NBP2-29467, Novus, USA), C/EBPδ (ab65081, Abcam, UK), CK12 (AP12735b, ABGENT, USA), CK3 (08691431, MP Biomedicals, USA) and Connexin 43 (C6219, Sigma-Aldrich, UK) in recommended dilutions overnight at 4 °C. An example of immunofluorescent staining is shown in Suppl. Fig. 1. Next day, the slides were washed three times with PBS for 5 min and then incubated with secondary antibody which was conjugated with FITC for 30 min in the dark at room temperature. An isotype control was used as a negative control where the primary antibody was omitted. Following this, cells were washed and then mounted in Vectashield anti-fading media containing Hoechst (Vector Laboratories, UK). Images were obtained with Zeiss Axio Imager (Carl Zeiss Microscopy, Germany). The images were analyzed with ImageJ by marking and counting the immunostained cells as well as total cells separately. A minimum of 300 cells per treatment were counted and the percentages of immunostained cells was calculated.

### 2.7 Quantitative reverse transcription polymerase chain reaction (qRT-PCR)

The Ambion Cells-to-cDNA™ II Kit (AM 1723, Life technologies, UK) was used for the isolation of total RNA and cDNA synthesis from cells of each zone according to the manufacturer's instructions. Quantitative RT-PCR is an accurate way of both detecting and quantifying a given DNA sequence and represents the most sensitive way of detecting differences in mRNA expression irrespective of exact cell number. The standard reference gene used for the studies below was *GAPDH*. The primers for qRT-PCR are listed in Table 1. Quantitative RT-PCR was performed using the LightCycler™ quantitative PCR machine (Roche, Switzerland). Each reaction was composed with 5 μl master mix, 3.7 μl RNase-free water, 0.4 μl forward and reverse primer mix, 0.8 μl template cDNA and 0.1 μl COX (all reagents from Promega, UK). Quantitative RT-PCR was performed using the LightCycler at 95 °C for 5 min, followed by 40 cycles of 94 °C for 15 s, primer specific annealing temperature for 30 s, and 72 °C for 20s, with a single data acquisition step. The crossing point for each transcript was determined using the LightCycler software (Roche, Switzerland). The LightCycler Relative Quantification software (Roche, Switzerland) was used to analyse the data. Each cDNA sample was measured in triplicate with *GAPDH* as the reference gene and using RNAse-free water as the negative control.

### 2.8 Statistical analysis

When more than two groups were compared, statistical analysis was carried out using one way ANOVA (Prism 3.0) with Welch's corrections. For comparison of two groups Student T-test analysis was used. Results were considered significant if p < 0.05.

## 3 Results

### 3.1 The growth rate, cell number and viability of limbal explant epithelial cells expanded on HAM in various minimal media

To assess the individual impact of adenine, cholera toxin, hydrocortisone and triiodothyronine, different media were prepared where each of these components was omitted and compared to the whole media containing all supplements. The explant outgrowths onto the HAM were observed in all the cases with cultures starting to grow from day 3–5 and reaching confluence by day 13–14 (Fig. 1A). Assessment of the epithelial outgrowths indicated similar growth rates with exception of media lacking adenine where an increased growth rate was observed (p < 0.05). Growth rate is measured as surface area and since it is possible that cells display different surface area depending on their state of differentiation, we assessed total cell numbers under all conditions (Fig. 1C). This analysis indicated a significantly decreased cell number in all three zones examined (A, B and C) when each component was omitted with a single exception in zone C where the cell number in media without hydrocortisone was similar to cell number obtained from explants grown in whole media. Assessment of cell size indicated a 10% increase in explants growing in the absence of adenine in zone A, which presumable accounts for the faster growth rate and yet reduced cell number in this zone (data not shown). Assessment of cell viability indicated similar survival except in media lacking triiodothyronine where a higher amount of dead cells was found in zone A and B and no cell grown in zone C, indicating a role for this component in maintaining cellular viability (Fig. 1D).

### 3.2 Colony forming potential of limbal explant epithelial cells expanded on HAM with various minimal media

**Fig. 2:**
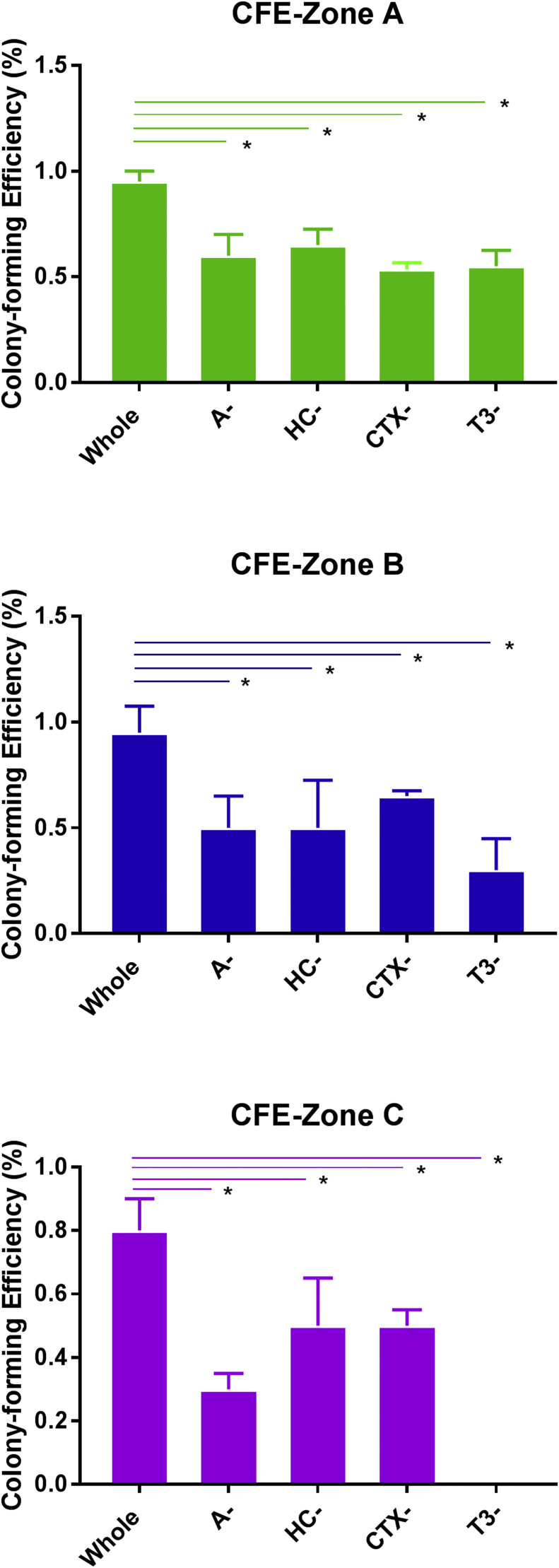
**The colony forming efficiency of *ex vivo* expanded limbal epithelial cells in whole and minimal media combinations**. Data are presented as mean ± SEM, n = 3, * p < 0.05.

Work carried out in our group has shown the highest concentration of progenitors in epithelial outgrowth next to the explant (zone A; Fig. 1B) when compared to the other two zones further away from the explant (B and C) (Kolli et al., 2008). We evaluated the impact of the four selected culture components and found that the CFE capacity decreased significantly across all zones in all four media lacking adenine, cholera toxin, triiodothyronine and hydrocortisone respectively (Fig. 2), indicating that each of these components is currently indispensable for the maintenance and proliferation of limbal epithelial progenitor cells.

### 3.3 Expression of putative limbal epithelial stem, progenitor and differentiated cells in limbal explant epithelial cells expanded on HAM with various minimal media

**Fig. 3:**
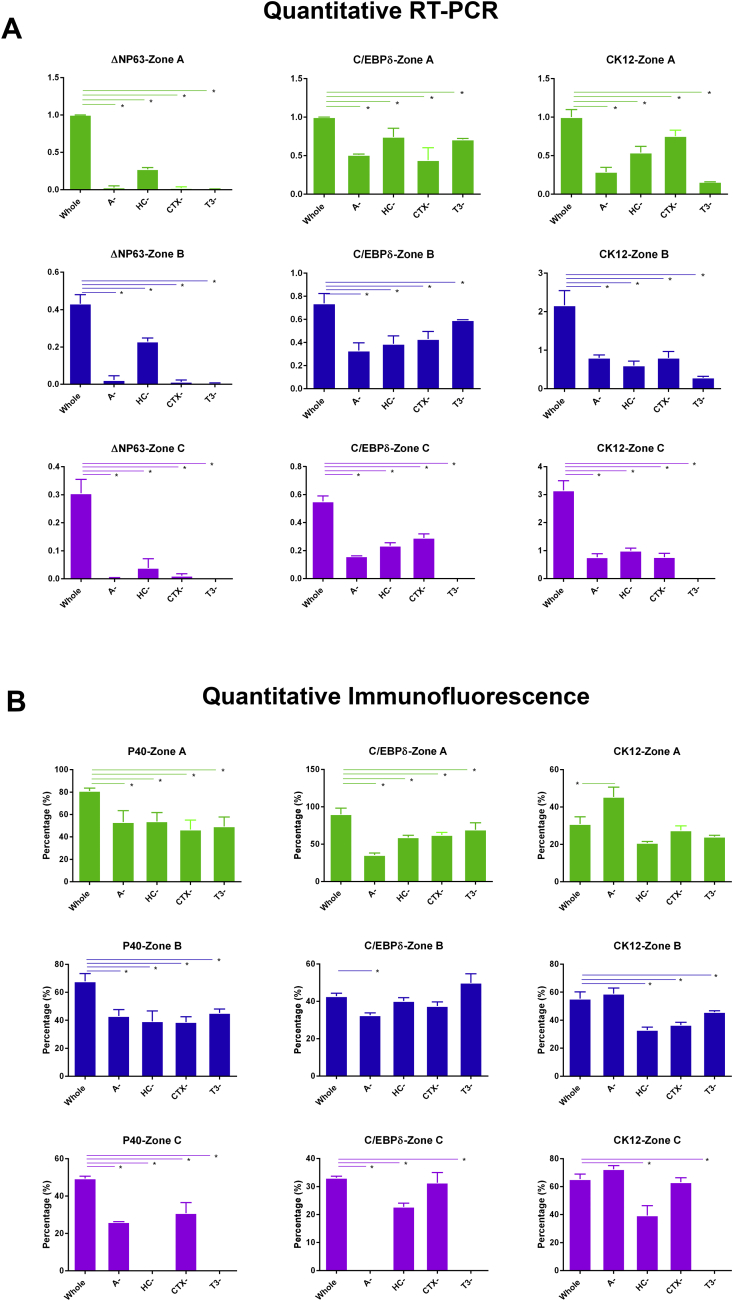
**Assessment of expression of putative LSC and corneal epithelial cell markers by qRT-PCR (A) and immunofluorescent staining (B) in whole and different minimal media combinations (B)**. Data are presented as mean ± SEM, n = 3, * p < 0.05.

**Fig. 4:**
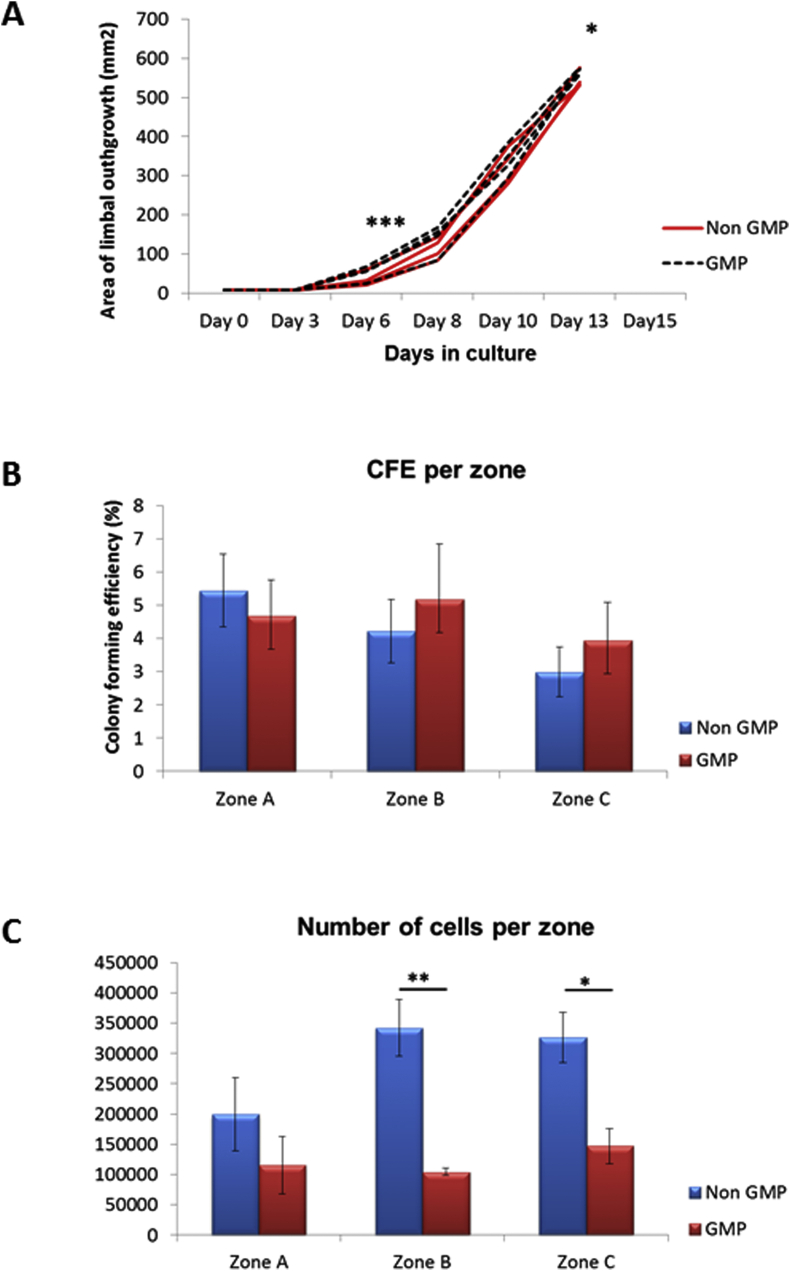
**Limbal epithelial cell growth, colony forming efficiency and cell numbers in non-GMP and GMP-grade media. (A)** Schematic graph showing the area of explant outgrowth (mm^2^) on different days of culture for the explants cultivated in non GMP (n = 4) and GMP-grade medium (n = 4); (**B**) The colony forming efficiency of cells from different zones of outgrowths cultivated in non- GMP (n = 4) or GMP-grade medium (n = 4) was performed as described in Section 2.5.; (**C**) Cell numbers per zone in non-GMP and GMP-grade media (n-4). Data are presented as mean ± SEM, * p < 0.05, ** p < 0.01, *** p < 0.001.

To assess the impact of individual media components on LSCs and differentiated epithelial cells, we carried out immunocytochemistry and qRT-PCR with three key markers: ΔNp63 (p40), C/EBPδ and CK12. Both ΔNp63 and C/EBPδ are known as putative LSC markers (Barbaro et al., 2007, Di Iorio et al., 2005, Pellegrini et al., 2001), however they are responsible for related but distinct processes: ΔNp63 is required for proliferation of LSCs, whereas C/EBPδ is required for their self-renewal. Our analysis indicated that the expression of ΔNp63 is significantly reduced both at the mRNA and protein level (Fig. 3A and B) when each of the four components are removed from the media. Quantitative RT-PCR analysis for the expression of C/EBPδ (Fig. 3A) indicated a significant downregulation of this LSC marker across all epithelial outgrowth zones whether they were close or far from the explant. However, immunocytochemistry results indicated significant downregulation of this marker in zone A in the absence of each component investigated (Fig. 3B) and in zone B and C in explants grown in media without adenine, suggesting that adenine exerts a significant impact in LSC self-renewal throughout the three epithelial outgrowth zones, whilst the other three components affect LSC self-renewal mostly in the outgrowth situated next to the explant. CK12 was used as a marker of differentiated corneal epithelial cells. The quantitative RT-PCR analysis displayed significant decreases throughout all zones (Fig. 3A), whilst immunocytochemistry indicated a significant increase in the expression of this marker in zone A of explants grown in the media lacking adenine which is also corroborated by increased cell size as indicated in 3.1 (Fig. 3B). We also noticed inconsistent expression of the LSC and corneal epithelial differentiation markers in zone C in explants growing in the absence of hydrocortisone and triiodothyronine (Fig. 4B) which may indicate the presence of other cell types that are not characterised by the expression of corneal epithelial markers. This is also corroborated by the absence of significant differences in cell size between all groups (data not shown) which is likely to suggest that other cell types and not differentiated limbal epithelial cells may be present in this zone when each of the four components is removed from the LSC media.

Together our data suggest that removal of each single component reduces proliferation of LSCs across all three epithelial outgrowth zones and their self-renewal in explant outgrowth closest to the explant. However two of the components namely adenine and triiodothyronine seem to play a more significant effect as removal of adenine results in increased percentage of differentiated corneal epithelial cells in the epithelial outgrowths close to the explant (zone A) whilst removal of triiodothyronine reduces the survival of epithelial cells across all outgrowth areas.

### 3.4 Growth rate, cell number and viability, CFE assay and expression of different markers in limbal explant epithelial cells expanded in GMP-grade medium

Results shown in previous sections indicated that each of the four investigated components were essential for maintaining appropriate LSC self-renewal and proliferation; hence we replaced each of those with GMP grade reagents in our GMP-grade media. Growth rate, cell number and viability, colony-forming efficiency together with expression of putative stem cell marker p40 (ΔNp63) and markers of corneal differentiation (cytokeratin 3 and connexin 43) were used to fully analyse the potential impacts of the new GMP media.

**Suppl. Fig. 2:**
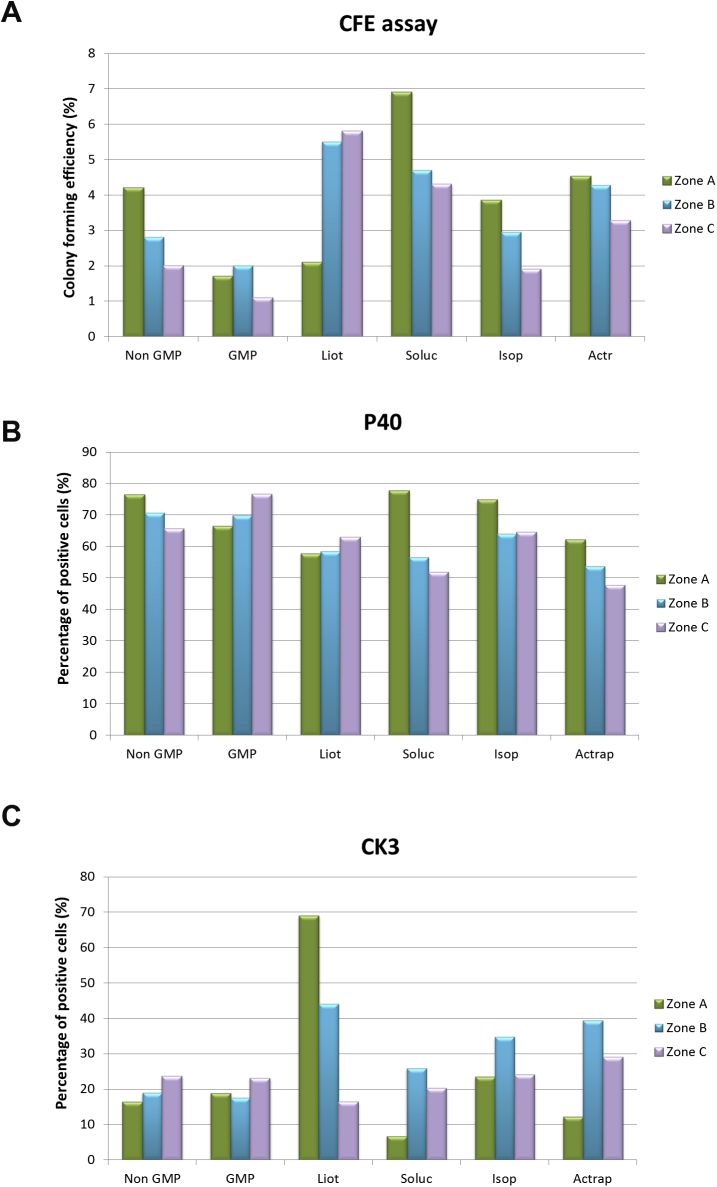
The colony forming efficiencies and expression of putative LSC and corneal epithelial cell markers cultivated in non GMP-grade medium, GMP-grade medium, medium with replacement of Liothyronine in isolation, medium with replacement of Solu-Cortef^®^ in isolation, medium with replacement of Isoprenaline in isolation and medium with replacement of Actrapid^®^ in isolation. (A) The colony forming efficiencies per zone of outgrowths cultivated in different media; (B) Expression of p40 per zone of outgrowths cultivated in different media; (C) Expression of CK3 per zone of outgrowths cultivated in different media and (D) Expression of Connexin 43 per zone of outgrowths cultivated in different media.

**Suppl. Fig. 3:**
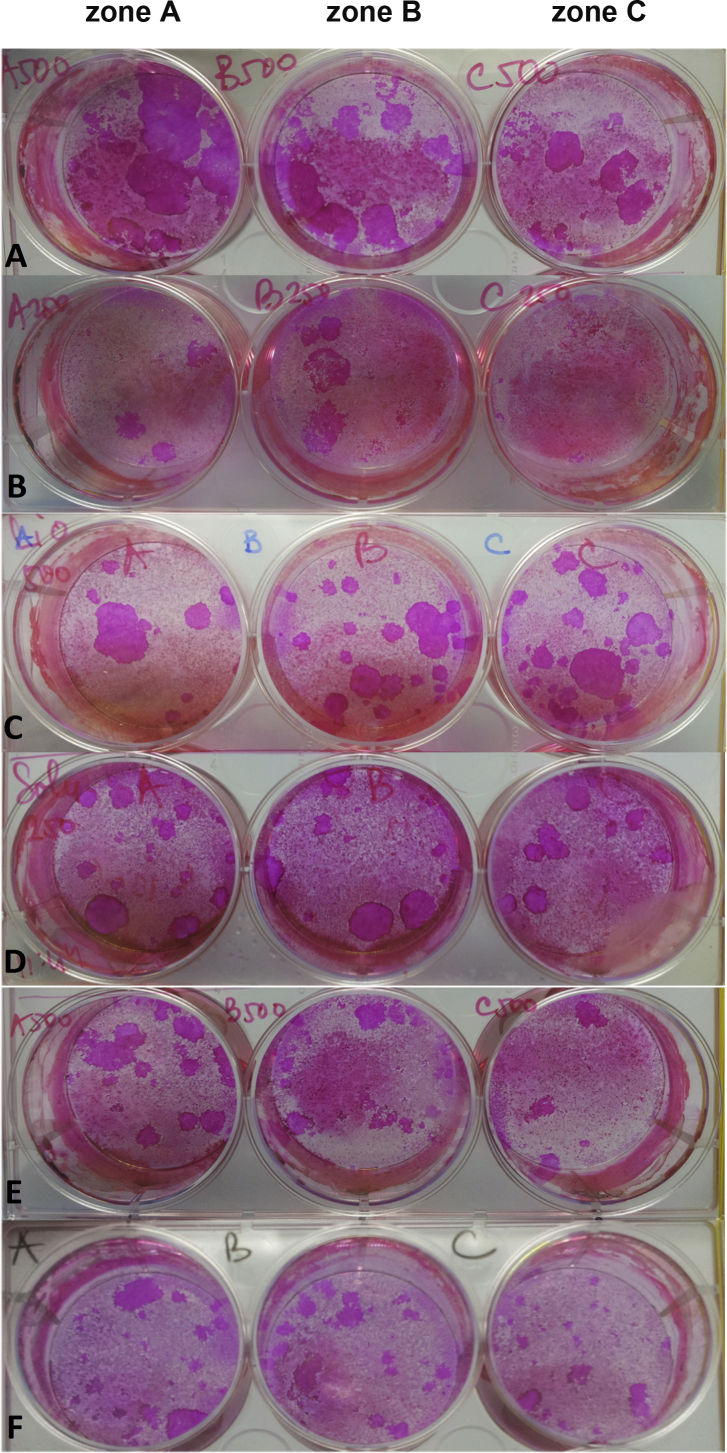
Appearance of colonies per zone of outgrowths cultivated in different media: (**A**) non GMP-grade medium; (**B**) GMP-grade medium; (**C**) medium with replacement of Liothyronine in isolation; (**D**) medium with replacement of Solu-Cortef^®^ in isolation; (**E**) medium with replacement of Isoprenaline in isolation; (**F**) medium with replacement of Actrapid^®^ in isolation.

**Fig. 5:**
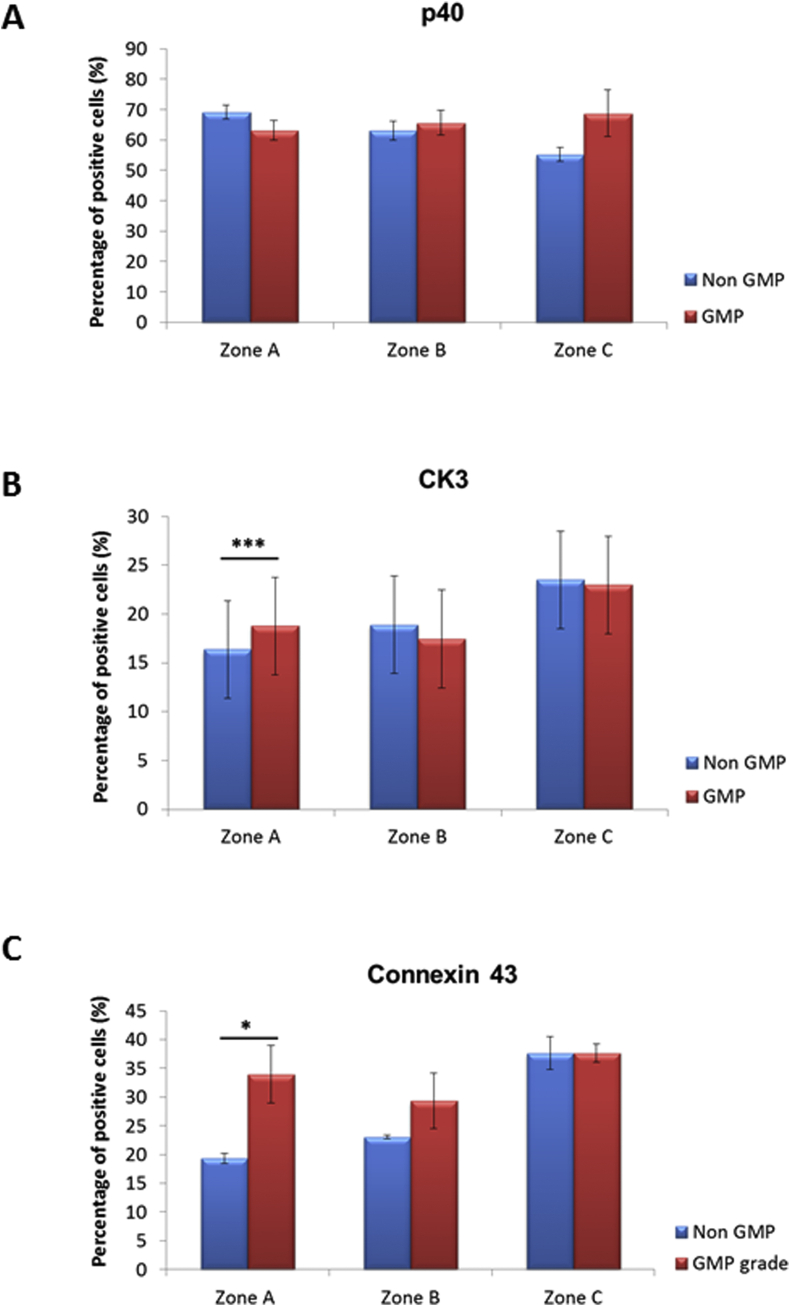
**Expression of putative LSC and epithelial cell markers in non-GMP and GMP-grade media assessed by immunofluorescent microscopy**. (**A**) Expression of ΔNp63 using the p40 antibody; (**B**) expression of CK3; (**C**) Expression of Connexin 43. (A–C) Data presented as mean ± SEM, n = 4, * p < 0.05, *** p < 0.001.

Successful cultures for explant outgrowths and CFE assay were obtained using both non GMP and GMP-grade media. 90% confluence was reached at day 13 in both groups. Growth rates, however, differed significantly between the groups at day 6 (p < 0.001) and day 13 (p < 0.05) with limbal epithelial cultures showing earlier onset of cell growth and greater growth area at the end of culture for explants cultivated in GMP-grade medium (Fig. 4A). Cell number in zone A was not significantly different whilst cell numbers in zones B and in zone C were significantly higher in the explants cultivated in non GMP-grade medium (Fig. 4C). Analysis of CFE (Fig. 4B) and percentage of p63 positive cells (Fig. 5A) didn't reveal any significant difference between the zones of outgrowths cultivated in non GMP and GMP-grade medium. The percentage of cytokeratin 3 (Fig. 5B) and connexin 43 positive cells (Fig. 5C) was significantly higher in zone A suggesting the presence of more differentiated cells in zone A of outgrowths cultivated in GMP-grade medium. Distribution of LSCs in non GMP-medium followed a characteristic pattern, previously demonstrated by our group, with the highest percentage of progenitors in zone nearest to the explant (zone A) with successive decline in zones B and C which are situated further away from the explant, whilst distribution of progenitors in GMP-grade medium did not follow this pattern (Fig. 5A). Analysis of every substituted component in isolation showed that Liothyronine may be responsible for the altered distribution of progenitor cells in zones A to C (Suppl. Fig. 2A-D and Suppl. Fig. 3).

## 4 Discussion

The aim of our study was to investigate the impact of adenine, hydrocortisone, triiodothyronine and cholera toxin on the *ex vivo* expansion of limbal explant epithelial cells on HAM. All these four components have been traditionally used in the skin and limbal epithelial explant cultures and shown to promote the growth and morphology of keratinocytes (Rheinwald and Green, 1975), the colony-forming ability of epithelial cells (Allen-Hoffmann and Rheinwald, 1984), proliferation of epithelial cells and the requirement for large amounts of fetal calf serum in the media (Allen-Hoffmann and Rheinwald, 1984, Kolli et al., 2010). However, to date there is little known of their individual roles in limbal stem cell culture.

Our data indicates that limbal epithelial explants could grow in media lacking any of the four selected components; however triiodothyronine stood out for its impacts on cell survival and adenine for increased explant surface area. A combination of CFE assays, immunocytochemistry and qRT-PCR for putative limbal stem and corneal epithelial cell markers indicated that all four components play a critical role in the proliferation and self-renewal of LSCs in all zones whether they were closer or further away from the explant. Our previous work has shown that self-renewal of LSCs, their migration and differentiation can be mimicked by our *ex vivo* culture system where the outgrowth zone nearer to explant contains the highest proportion of progenitor cells and as they move away from the explant they differentiate to acquire features typical of corneal epithelial cells (Kolli et al., 2008). In accordance with this, removal of each of the four components reduced the self-renewal of LSCs in the outgrowth nearest to the explant. Furthermore, the removal of adenine also led to increased percentage of differentiated cells in the zone closest to the explant as well as increased cell size. Together our data suggest that all four components are currently indispensable for the successful *ex vivo* expansion of limbal epithelial explants on HAM and for maintaining the highest number of proliferating LSCs.

The balance between self-renewal and differentiation of LSCs is tightly regulated *in vivo* with LSCs moving centripetally towards the centre of the cornea and differentiating to give rise to the corneal epithelial cells which are replaced approximately every 14 days (Thoft and Friend, 1983). At the same time, the limbal stem cell niche maintains the self-renewal of LSCs through uneven distribution of cell fate determinants across the corneal epithelium. ΔNp63α and C/EBPδ have been shown in asymmetric cell division and early cell fate decision of human limbal stem cells (Mort et al., 2012). Increasing evidence supports the theory that p63 promotes the maintenance of LSCs and also is a determinant of their proliferative potential. Pellegrini et al. has reported that the number of p63 bright cells is an important prospective measure of determining the clinical success of LSC transplantation (Pellegrini et al., 2011). Our LSC culture system on HAM results in generation of a high percentage of p63 + cells, namely: 78.1% in zone A, 67.8% in zone B and 49.4% in zone C; however the number of p63 + cells significantly decreased to nearly half of that from standard medium when treated with media lacking any one of the four components tested in this study, thus suggesting that removal of each of these four components may pose a risk for clinical translations.

Therapeutic use of LSCs must be performed in compliance with Good Manufacturing Practice as a quality assurance system to ensure highest quality and safety of cell product for transplantation in accordance with the European Union, Regulation (EC) No 1394/2007 of the European Parliament and of the Council, on Advanced Therapy Medicinal Products. As part of our study, we aimed at replacing all ingredients that were not produced according to GMP guidelines with GMP-grade products. This is a rather important safety aspect as it has been shown that animal-derived components can cause severe immunologic reactions and potential transmission of microorganisms (Erickson et al., 1991, Selvaggi et al., 1997, Chachques et al., 2004, Schwab et al., 2006). We chose widely used clinical grade products Solu-Cortef^®^, Actrapid^®^, Liothyronine or Isoprenaline to replace non-GMP grade ingredients. Isoprenaline β-adrenergic agonist is commonly used for the treatment of bradycardia and is known to increase intracellular calcium concentration of bovine corneal epithelial cells by inducing stimulation of cyclic AMP and thus it represents a safe alternative to cholera toxin for LSC cultivation (Reinach et al., 1992, Akhtar and Choi, 1994). CTX and L-isoproterenol are largely used to enhance cell proliferation (Ghoubay-Benallaoua et al., 2012). It was particularly important to replace cholera toxin since this is obtained from bacteria cultured on bovine brain broth and fetal calf serum. While no data have been published for Solu-Cortef^®^ and Liothyronine, Actrapid^®^ and Isoprenaline were previously used for LSC cultivation with success (Ghoubay-Benallaoua et al., 2012). These previous data indicated that isoprenaline-supplemented medium (2 μg/ml) is more efficient than cholera toxin for enhancing cell growth and decreasing cell size in two-week cultures (Ghoubay-Benallaoua et al., 2012). In accordance with this, we observed a slightly faster growth rate of explants in the GMP culture media and a larger growth area at the end of culture period. Although CFE per zone did not differ significantly between the groups, the morphology of colonies was different. Cells cultivated in the GMP-grade medium formed colonies with more differentiated appearance compared with cells cultivated in the non GMP-grade medium. While the percentage of progenitor cells per zone did not differ between groups, distribution of progenitor cells per zone compared with explants cultivated in non GMP-grade medium was unexpectedly altered. Furthermore, it seems that cultivation in GMP-grade medium promoted differentiation of cells in the zone nearest to the explant. Analysis of every substituted component in isolation showed that Liothyronine may be responsible for the altered distribution of progenitor cells in zones A to C and for promoting the differentiation of progenitor cells in zone A. Notwithstanding these differences, LSCs expanded in LSC media with GMP grade reagents have not been tested *in vivo*. With this in mind, it would be useful to test their potential to engraft and reverse LSCD in animal model settings (for example rabbit) to investigate whether the molecular differences we have observed *in vitro* hold true *in vivo*.

## 5 Conclusion

Our data suggests that all four components were essential for maintaining LSC proliferation across all three epithelial outgrowth zones and LSC self-renewal in the outgrowth zone situated closest to the explant. Replacement of each of the LSC media components with GMP grade reagents resulted in equal growth to non-GMP grade media; however an enhanced differentiation of progenitor cells in the outgrowth situated closest to the explant was observed, suggesting that additional combinations of GMP grade reagents need to be tested to achieve similar or better level of LSC maintenance in the same manner as the traditional LSC media. The research strategy of better refining the current stocks of materials and their possible replacement with GMP grade components provides a pathway that may be beneficial to other medicinal advanced cell therapy products currently being used in clinical trials.

## Figures and Tables

**Table 1:** DNA oligonucleotides used in qRT-PCR analysis.

Name of primer	Sequences (5′-3′)
*GAPDH-F*	GTC AGT GGT GGA CCT GAC CT
*GAPDH-R*	CAC CAC CCT GTT GCT GTA GC
*ΔNp63-F*	CTG GAA AAC AAT GCC CAG AC
*ΔNp63-R*	GGG TGA TGG AGA GAG AGC AT
*C/EBPδ-F*	CCTCCCAAAATGCTGGGATTAC
*C/EBPδ-R*	TCCAGGTCTACGGAAGCAGTG
*CK12-F*	GAA GAA GAA CCA CGA GGA TG
*CK12-R*	TCT GCT CAG CGA TGG TTT CA
